# Editorial: Cardio-circulatory support of neonatal transition

**DOI:** 10.3389/fped.2023.1146395

**Published:** 2023-02-13

**Authors:** Bernhard Schwaberger, Gerhard Pichler, Nariae Baik-Schneditz, Stefan Kurath-Koller, Hannes Sallmon, Yogen Singh

**Affiliations:** ^1^ Division of Neonatology, Department of Pediatrics and Adolescent Medicine, Medical University of Graz, Graz, Austria; ^2^ Division of Pediatric Cardiology, Department of Pediatrics and Adolescent Medicine, Medical University of Graz, Graz, Austria; ^3^ Department of Pediatric Cardiology, Charité-Universitätsmedizin Berlin, Berlin, Germany; ^4^ Department of Pediatrics - Neonatology and Pediatric Cardiology, Cambridge University Hospitals NHS Foundation Trust and University of Cambridge School of Clinical Medicine, Cambridge, United Kingdom; ^5^ Department of Pediatrics - Division of Neonatology, Loma Linda University Children’s Hospital and Loma Linda University School of Medicine, Loma Linda, CA, United States

**Keywords:** neonatal stabilization, neonatal transition, cardio-circulatory monitoring, cardio-circulatory support, neonatal resuscitation and care, timing of cord clamping

**Editorial on the Research Topic** Cardio-circulatory support of neonatal transition

## Introduction

Immediate transition from fetal-to-extrauterine life is accompanied by complex physiological processes affecting all vital organs including the cardio-circulatory system. In the fetus, pulmonary vascular resistance is high, and most of the right ventricular output bypasses the lungs *via* the ductus arteriosus into the systemic circulation. Following the elimination of the low-resistance placenta by clamping the umbilical cord and the decrease of pulmonary vascular resistance with the first breaths after birth, major hemodynamic changes occur. The consecutive fall in pulmonary vascular resistance and increase in systemic vascular resistance further induces the reversal of ductal shunting from right-to-left to left-to-right, with functional closure of the ductus arteriosus within 24–48 h after birth in healthy term infants (1, 2).

### Timing of cord clamping

Clamping of the umbilical cord eliminates placental circulation, and switches gas exchange to the lungs. Although the procedure of umbilical cord clamping is quick and simple, the timing may have a substantial impact during the immediate neonatal transition period (3). Recent research focused on the appropriate timing of the intervention, distinguishing immediate from deferred umbilical cord clamping (3–5). By deferring the time of cord clamping, the blood flow between the placenta and the neonate continues up to five minutes (6), potentially leading to “placental transfusion” that will increase hemoglobin levels, cardiac output, and thereby oxygen delivery (3, 7). When the umbilical cord is clamped before lung aeration, left ventricular preload and cardiac output decrease significantly, as preload mainly depends on umbilical venous return *via* the ductus venosus, while pulmonary venous return has not yet increased. Thus, left ventricular preload remain low until lung aeration is established and pulmonary blood flow increases (8). Therefore, “physiological-based cord clamping” (PBCC) was introduced offering the neonate time to establish spontaneous breathing while still being placentally supported (9). Whereas deferred cord clamping is a time-based approach (e.g., >1 min after birth), PBCC is defined as clamping the umbilical cord after aeration of the lungs (9). Schwaberger et al. investigated two different cord clamping strategies in healthy full-term neonates. They demonstrated no significant differences in cerebral tissue oxygenation and cerebral blood volume during the first 15 min after birth, irrespective of whether cord clamping was performed after the onset of stable regular breathing at 275 s (IQR: 197–345 s) after birth (intervention group) or according to the standard procedure at 58 s (IQR: 35–86 s) after birth (control group). The authors concluded that deferring cord clamping ≥1 min following a physiological-based approach offered no additional benefits regarding cerebral oxygenation and perfusion compared to the routinely used time-based cord clamping approach in a group of healthy neonates after uncomplicated vaginal delivery. Further studies focusing on PBCC in different populations such as neonates requiring initial respiratory support or with adverse conditions such as prematurity, asphyxia, or sepsis are warranted.

### Cardio-circulatory monitoring

Besides clinical assessment and routine monitoring (pulse-oximetry and/or electrocardiography), additional cardio-circulatory monitoring may offer further insights into cardiovascular transition (10). Ideally, this assessment should be non-invasive and provide reliable information continuously. Recent research focused on non-invasive cardiac output monitoring (NICOM), and the assessment of end-organ perfusion by using near-infrared spectroscopy (NIRS) (2, 11). Berisha et al. performed a feasibility study using impedance cardiography for NICOM in asphyxiated piglets to evaluate its potential to improve monitoring and treatment of sick neonates. Half of the registrations in asphyxiated piglets were of adequate quality, albeit signal quality was highly variable between individuals. The presented data was interpreted to be physiologically plausible and support further research evaluating this method for NICOM in clinical studies. The signal quality was superior to recently published delivery room studies in human neonates using electrical velocimetry for NICOM in which about ¾ of the registrations were excluded due to poor signal quality (12, 13). However, in the study by Berisha et al. the measurements were conducted in sedated animals under strictly controlled laboratory conditions which doesn’t reflect the clinical situation in the delivery room. Pfurtscheller et al. performed a post-hoc analysis of prospective observational studies in which NIRS monitoring during neonatal transition after birth was used. They assessed whether arterial blood pressure was associated with cerebral regional oxygen saturation and cerebral fractional tissue oxygen extraction in preterm neonates with and without respiratory support. In compromised moderate and late preterm neonates receiving respiratory support after birth, they found a significant correlation between NIRS derived parameters and arterial blood pressure. These findings suggest that passive pressure-dependent cerebral perfusion was present, indicating an impaired cerebral autoregulation in those compromised preterm neonates during the immediate transition. Those upcoming techniques require further investigation in clinical trials as they potentially identify neonates in need of cardio-circulatory support, and further guide hemodynamic management during immediate fetal-to-neonatal transition (2, 10, 11).

### Cardio-circulatory support

Once neonates in need of cardio-circulatory support are identified, establishing vascular access, administering emergency drugs and fluid therapy (e.g., to treat or avoid arterial hypotension), and performing chest compressions in cases of severe bradycardia are vital interventions during the immediate neonatal transition (14–16). The hemodynamic management immediately after birth may have a impact on the ongoing transition from fetal-to-neonatal circulation during the first days and weeks after birth. In this context, the absence of physiologic closure of the ductus arteriosus and the therapeutic regimen of this condition is a “hot topic”, especially in premature infants (17–19). If the ductus arteriosus remains patent after one or two weeks, it may cause severe left-to-right shunting and overloaded pulmonary perfusion resulting in pulmonary edema, pulmonary hypertension, and bronchopulmonary dysplasia (18). Ho et al. published a literature review about the pathophysiology of patent ductus arteriosus, and some *in silico* models used for blood flow and arterial wall modeling. They stress that a multiscale paradigm needs to be adopted when developing future *in silico* models for patent ductus arteriosus to optimize treatment strategies.

During immediate neonatal transition, optimally timed umbilical cord clamping, improved cardio-circulatory monitoring and properly applied cardio-circulatory support have the potential to reduce mortality and morbidities, while suboptimal care could cause long-term sequelae. The research topic may contribute to consolidating the current body of information with the hopes of enhancing and stimulating further studies on all spectra of “Cardio-Circulatory Support of Neonatal Transition”.
